# Demographic features and clinical characteristics of patients with Parkinson’s disease in Isfahan, Iran

**Published:** 2018-01-05

**Authors:** Mehri Salari, Omid Mirmosayyeb, Masoud Etemadifar, Vahid Shaygannejad, Fariborz Khorvash, Mohammad Reza Najafi, Fereshteh Ashtari, Ahmad Chitsaz

**Affiliations:** 1 Isfahan Neurosciences Research Center, Department of Neurology, School of Medicine, Isfahan University of Medical Sciences, Isfahan, Iran; 2 Student Research Committee, Isfahan University of Medical Sciences, Isfahan, Iran

**Keywords:** Parkinson Disease, Demographic, Iran

## Abstract

**Background:** Parkinson’s disease (PD) is the second most common neurodegenerative disorder. Its worldwide incidence rate varies between 18 and 418 cases per 100000 annually. This cross-sectional study was carried out with the aim to identify the clinical characteristics and demographic features of a huge number of patients with PD in Isfahan, Iran.

**Methods:** The study was conducted on 987 patients with PD in Isfahan city and checklists were used to evaluate the demographic features and clinical characteristics of the patients.

**Results:** The mean age of the patients at the time of sampling was 65.40 ± 11.90 years. The study results indicated that the rate of PD among men with 67.3% was twice that of women with 32.7%. The mean duration of the disease was 4.91 ± 4.60 years.

**Conclusion:** This study showed a considerable rate of PD among the individuals in Isfahan city. In addition, the incidence ratio of men to women was more than the previous studies.

## Introduction

Parkinson’s disease (PD) is the second most common neurodegenerative disorders.^1^ The worldwide incidence rate of this disease varies from 18-418 to 5-20 cases per 100000 according to the studies by Konitsiotis, et al.^2^ and Jankovic and Tolosa^3^, respectively. The differences could be due to the different methodologies, diagnostic criteria, and variation in populations.

A systematic review in Asia using door-to-door surveys showed standardized all-age incidence rates of 51.3 to 176.9 reported cases per 100000 and 8.7 reported cases per 100000, respectively. The incidence rate in record-based studies ranged from 35.8 to 68.3 reported cases per 100000 and 6.7 to 8.3 reported cases per 100000.^4^

There are no reports on the incidence of PD in Iran. This cross-sectional study was conducted aiming to identify the demographic features and clinical characteristics of a huge number of patients with PD in Isfahan, Iran. The age of onset, family history, past medical history, and Parkinsonism symptoms have been reported in this study.

## Materials and Methods

Isfahan Province is a large area located in the center of Iran and is 1590 meters above sea level with coordinates of 32.6577 ∞° N 51.6692 ∞° E. The total area of the Province is 107 km^2^ and its climate is dry with a mean daily temperature of 5.3 °C in January and 27.2 °C in August. Based on the census conducted in 2015, its population was 5 million, 37.5% of which were living in Isfahan city. The present study was conducted in Isfahan city with an area of 550 km^2^. The age of 13.4% and 6.5% of the population of this city was between 50 and 64 and above 65 years old, respectively. 

This study was conducted from December 2014 to January 2016. Two sources were used to collect patients with PD: two referral hospitals equipped with movement disorders clinics, and neurologists who visited patients in Isfahan city. The neurologists in this study were experienced in movement disorders and diagnosed PD based on the United Kingdom Parkinson’s Disease Society Brain Bank Criteria (UKPDSBBC) for idiopathic PD. Genetically transmitted PD was also included. The exclusion criterion was patients who were unwilling to participate in the study. The written informed consent was obtained from the patients. In addition, the study was approved by the ethics committee of Isfahan University of Medical Sciences with the code 395322.

During the study, each patient who fulfilled the criteria was interviewed and the information including, age, gender, job, education, birthplace, age of onset, and disease durations was collected by the checklists.

A family history of PD among the first to third-degree relatives was obtained from each patient and the exact relationship to the patient was determined. In a positive family history, other questions were asked, including symptom type, medications used, response to treatment, and symptoms compatible with atypical Parkinsonism, to avoid false positive responses.

In order to identify smoking habits, questions were asked about previous or current habits. Subjects were also encouraged to provide information about comorbid disease that affected them including hypertension, diabetes mellitus (DM), benign prostatic hypertrophy (BPH), cardiovascular diseases (CVDs), cancers, thyroid dysfunction, and renal impairments. 

The neurologists recorded clinical disease information like presence of tremor, rigidity, bradykinesia, freezing, and instability. In case of a speech or memory problem, family members or caregivers of the patients were interviewed to collect information.

All data were analyzed using the SPSS software (version 23, IBM Corporation, Armonk, NY, USA). Quantitative demographic characteristics were expressed as mean ± standard deviation (SD) and qualitative data have been shown as a percentage. The Student’s t-test and Mann-Whitney U-test were used to compare means of the data with normal and abnormal distributions, respectively. Moreover, the chi-square and Fisher’s exact tests were used to compare the correlations between the two groups. A P value of < 0.050 was considered statistically significant.

## Results

**Figure 1:**
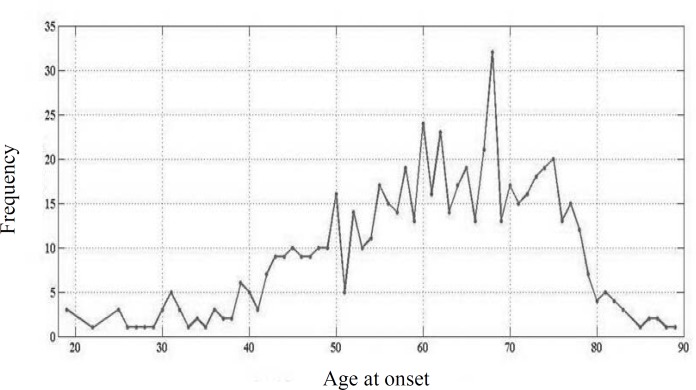
Rate of patients in terms of age at onset

A total of 987 patients diagnosed with PD were included in the study. The mean age of patients at the time of sampling was 65.40 ± 11.90 years with a range of 21 to 92 years. The results of the study indicated that the rate of PD among men with 67.3% was twice that of women with 32.7%. In addition, the mean duration and the self-reported age of onset of the disease was 4.91 ± 4.60 and 60.84 ± 13.00 years with a range of 19 to 89 years, respectively. 136 patients (13.78%) had early onset of PD, denoted by diagnosis at 50 years or earlier; figure 1 demonstrates the rate of patients with regard to age of onset. There were no statistically significant differences between the disease duration and the age of onset. Moreover, no statistically significant differences were observed between men and women regarding the diagnosis age and the disease duration.

**Figure 2:**
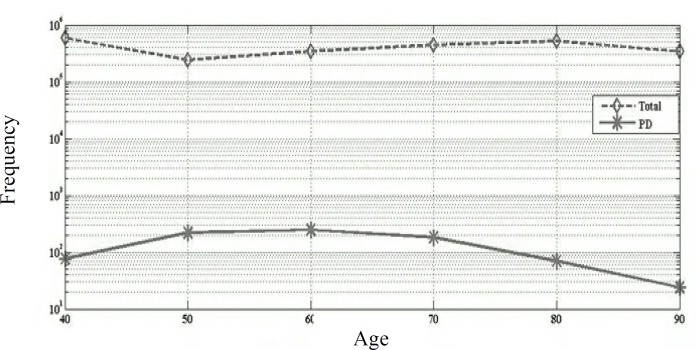
Rate of patients in terms of age

As shown in figure 2, most of the patients (30.2%) were between 61 to 70 years of age. In terms of education, 40.7%, 30.1%, 15.7%, and 13.6% of the patients were illiterate, graduated from elementary or junior high school, high school graduates, and with academic degrees, respectively. A history of smoking was reported among 12.6% of the patients. In addition, a family history of PD including one or more first to third-degree relatives was found among 91 (9.2%) of the patients.

Concerning comorbidities, 14.3%, 9.0%, 5.0%, 2.3%, and 1.2% of the patients were with diseases including hypertension, DM, CVDs, benign prostate hypertrophy, and a history of cerebrovascular accidents, respectively. Tremor with a rate of 74.1% was the most frequently reported symptom followed by bradykinesia, rigidity, instability, and freezing with rates of 73.5%, 56.8%, 49.1%, and 43.5%, respectively. There was no statistical difference between tremor, bradykinesia, rigidity, instability, or freezing and gender. However, there was a correlation between instability and age (P = 0.014) and freezing and age (P = 0.043). 

Regarding cigarette smoking, there was a strong direct correlation between smoking and tremor (P < 0.001). In addition, freezing and rigidity were correlated with smoking (P = 0.008 and P = 0.023, respectively). Based on these findings, the rate of PD was 47.0 reported cases per 100000; by gender division, this rate was 60 and 29 men and women per 100000 cases, respectively.

## Discussion

The present study is the first cross-sectional study in Isfahan city. Roohani, et al. reported the demographic characteristics of 1656 Iranian patients with PD,^5^ however, there are no other provincial studies on the rate of PD in Iran. In the present study, all patients referred to the neurologists were examined by a movement disorder specialist, which was one of the advantages of the study. Therefore, patients with atypical Parkinsonism were excluded.

The men to women ratio among the subjects in the present study was 2.06:1, which was higher than most of the rates reported in the studies worldwide. Previous studies reported men to women ratios of 1.56:1 in South America and 1.21:1 in Europe, North America, Australia and Asia.^6^ Roohani, et al. reported a ratio of 2.00:1 among the 1656 Iranian patients which was similar to the present study.^5^

The mean age of the patients was 65.40 ± 11.90 years in the present study, which was almost similar to Iranian population with a mean age of 65.10 ± 12.00. In a study conducted by Konitsiotis, et al., the mean age of the participants at the time of sampling was 70.40 ± 9.80 years, which was higher than that of the subjects in the present study.^2^

Most of the participants were referred from neurology clinics and offices. About 76.0% of the participants lived in Isfahan city center, hence, sampling was carried out among patients with high access to healthcare centers. The PD rate was estimated to be 47.0 reported cases per 100000 which is similar to the real rates. Therefore, a door-to-door study is required to determine the exact rate of PD in Isfahan city due to the worldwide diverse incidence of 10.0 to 405.0 cases per 100000. The highest rate of PD was among the patients in age range of 61-70 years as 248 reported cases per 100000 (Table 1), however, the worldwide PD rate of 1903 reported cases per 100000 is more predominant after the age of 80 years.^6^

**Table 1:** Parkinson’s disease (PD) rate among different age groups

**Age group (years)**	**Rate [n (%)]**
40 and younger	24 (2.4)
41-50	70 (7.1)
51-60	183 (18.5)
61-70	248 (25.1)
71-80	220 (22.3)
81 and older	77 (7.8)
Missing	165 (16.7)

As table 1 shows, the incidence of tremor increases with age, moreover, even higher incidence of PD was reported among individuals > 80 years old in almost all regions of the world.^6^ However, the PD rate decreased after 70 among the subjects of the present study, which may be due to the severe disability because of dementia and immobility in this age group, causing their lower referral compared to other age groups. In addition, it may be due to the low life expectancy among the population in Isfahan city. Decreased rate of PD in the oldest age groups has been reported in the previous studies as well.^7^^-^^11^

Family history was found to be positive among the 9.2% of participants in the present study. However, other studies have shown that 10.0 to 20.0% of patients have a positive family history. The lower PD rate in the present study compared to other published studies might be due to the undiagnosed cases among the subjects of this study.^12^^-^^14^

In terms of smoking, 12.6% of the subjects were smokers, which is within the range of 7.0–34.4% reported in other.^15^ In addition, there are evidences showing that smoking may delay the onset of PD.^16^

Some studies reported hypertension as the most incident cardiovascular risk factor among PD patients.^2^ Incidence rate of hypertension among the patients with PD is 26.2% in Iran, indicating a lower rate due to baroreflex and sympathoneural denervation as part of the disease course and dopaminergic drug use.^17^ The incidence of DM type II in Isfahan was reported as 2.3% to 9.3%; this rate was 9.0% among patients with PD in the present study.^18^ Based on the reports, DM might increase the risk of PD in the future.^19^

## Conclusion

This study was conducted in Isfahan for the first time using a large population of patients with PD. However, the data were only collected from neurology clinics, hence, door-to-door studies are required to determine the exact incidence of PD in Isfahan Province and Iran. 

Therefore, this study is the first to describe the clinical profile of a large population of patients with PD in Isfahan Province. Determining the diagnosis of PD by neurologists specialized in movement disorders validates this study. The researchers tend to conduct a comprehensive door-to-door country based study in order to determine the exact incidence of PD in Iran. As the Iranian population becomes older, the incidence rate of PD is expected to increase in the future, requiring optimal planning for healthcare and facilities for these patients.

## Notes:

**How to cite this article:** Salari M, Mirmosayyeb O, Etemadifar M, Shaygannejad V, Khorvash F, Najafi MR, et al. Demographic features and clinical characteristics of patients with Parkinson’s disease in Isfahan, Iran. Iran J Neurol 2018; 17(1): 6-10.
